# Novel Intraoperative Augmented Reality-Guided Specimen Localization in Chest Wall Oncologic Surgery: A Case Report

**DOI:** 10.1177/29941520261426873

**Published:** 2026-03-10

**Authors:** Andreja Radevic, Joaquin Austerlitz, Riley Gilbertson, Sakshi Krishna, Michael Colello, Daniel Johnson, Reena Singh, Alexander Perez, Joshua M. Lawrenz, Michael C. Topf

**Affiliations:** 1 Department of Otolaryngology-Head and Neck Surgery, Vanderbilt University Medical Center, Nashville, Tennessee, USA.; 2 Vanderbilt University School of Medicine, Nashville, Tennessee, USA.; 3 Department of Orthopedic Surgery, Vanderbilt University Medical Center, Nashville, Tennessee, USA.; 4 Department of Pathology, Microbiology and Immunology, Vanderbilt University Medical Center, Nashville, Tennessee, USA.

**Keywords:** augmented reality, chest wall reconstruction, 3D modeling, surgical margin localization, oncologic surgery, specimen mapping

## Abstract

Localization and communication of surgical margins remain a challenge in oncologic surgery, particularly in the setting of staged reconstruction where wound bed deformation alters native anatomical landmarks. This proof-of-concept case report evaluates the feasibility of augmented reality (AR) to reorient a specimen-derived 3D tumor model within the resection bed during staged chest wall reconstruction.

One patient with a recurrent undifferentiated pleomorphic sarcoma of the chest wall underwent radical chest wall excision with staged reconstruction. Following resection, the specimen was scanned with a structured-light 3D scanner to create a digital twin. The 3D model was virtually annotated to mirror the standard grossing process using a custom computer-aided design software. The mapped model was uploaded into an Apple Vision Pro (AVP) AR headset. On the day of the staged reconstruction, the holographic specimen model was reoriented *in situ* using predefined anatomical landmarks for registration during the surgical setup period. First-time AR users were able to manipulate and correctly position the model after a brief onboarding. AR-guided placement was completed without altering routine care or delaying operative workflow (surgeon onboarding to AVP: 5 min; AR specimen placement and verification: 5 min). Alignment was qualitatively confirmed with inked specimen surfaces and resected rib orientation in the wound bed. No positive margin was identified in this index case, therefore performance for margin relocalization was not assessed.

In this single-case study, AR-guided reorientation of a specimen-derived 3D model in reconstructive chest wall surgery was feasible and compatible with surgical workflows.

## Introduction

Achieving negative margins in oncologic surgery is imperative to minimizing the risk of recurrence and ensuring favorable oncologic outcomes.^1,2^ In the setting of a positive margin, accurate assessment of margin location on the resected specimen is critical for adequate wound bed reresection and achieving final margin-free status.^3^ However, orienting resected anatomy to the wound bed days later is challenging, as the wound bed may become deformed in the early postsurgical period.^4^

Pathological findings, such as positive margins, are typically conveyed to the surgeon through a written pathology report without any visual aid. The surgeon must then correlate these results, without visual aid, to the wound bed and reresect in cases of close or positive margins. This process is limited by both pathologist–surgeon communication gaps, as well as surgeon discrepancies, such as poor concordance in identifying soft tissue landmarks.^5–7^ Previous studies have demonstrated the feasibility and accuracy of 3D scanning and virtual annotation of resected cancer specimens to improve pathologist–surgeon communication of histological findings of resected cancer specimens.^8,9^ However, in the context of staged reconstruction, where wound bed deformation and communication gaps complicate specimen and wound bed orientation, there remains a need for innovation to enhance surgeons’ spatial understanding of tumor bed anatomy.

In this study, we describe the feasibility of generating a 3D virtual model of a resected chest wall sarcoma specimen, digitally annotating the grossing process, and uploading the model to an augmented reality (AR) headset. We then guided the orthopedic oncologic surgeon, a first-time AR user, through manipulation and repositioning of the model into the wound bed prior to staged reconstruction. We further evaluate the potential of AR to aid spatial comprehension of surgical anatomy and guide reconstructive planning.

## Case Presentation

Vanderbilt University Medical Center Institutional Review Board (IRB) approval was obtained for the 3D-scanning and annotation of resected cancer specimens as well as uploading 3D models to AR headsets (IRB # 250834).

A 71-year-old male with a history of left chest wall undifferentiated pleomorphic sarcoma treated with multiple prior resections and adjuvant radiotherapy 1 year prior was referred to our institution with a mildly tender left lateral chest wall mass adjacent to his prior resection site. Magnetic resonance imaging revealed an 8.8 cm *×* 5.6 cm nodular area of enhancement along the left lateral chest wall, abutting the ninth rib, suspicious for recurrence. Biopsy confirmed recurrent high-grade undifferentiated pleomorphic sarcoma. After a multidisciplinary tumor board discussion, the decision was made to undergo salvage surgery. The patient underwent radical resection of the tumor, including the left chest wall and ninth rib, with planned staged reconstruction 4 days postresection. Wet-to-dry dressings and an abdominal binder were used to dress the defect prior to reconstruction. The intention of generating the specimen-derived 3D model was to enable AR-guided reorientation of the resection specimen into the wound bed at the time of staged reconstruction and to provide a framework for targeted margin relocalization should final pathology identify a close or positive margin requiring additional reresection. From a feasibility standpoint, this case was also used to evaluate whether specimen scanning, virtual annotation, and AR-guided reimplantation could be performed without disrupting standard pathology or operative workflows.

### 3D scanning

**FIG. 1.**
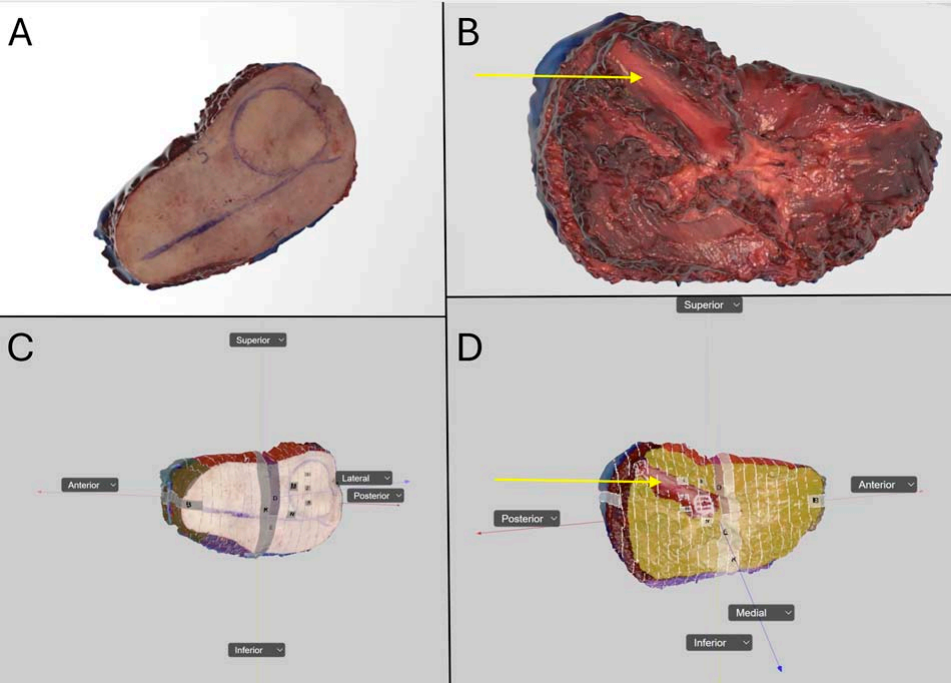
**(A–D):** Raw and mapped 3D models of resected chest wall specimen. **(A)** Raw scan, skin surface of resected specimen, in anatomical position. Intraoperative marking seen on skin surface denotes orientation; S = Superior, I = Inferior, P = Posterior. **(B)** Raw scan, deep/medial surface of the resected specimen, with the resected ninth rib seen on the superior–posterior aspect of the specimen (yellow arrow). **(C)** Mapped specimen, skin surface, mirroring the grossing process, including inking schema, cut sections, submitted sections with lettering, and axes labeling orientation. **(D)** Mapped specimen, deep/medial surface, with same annotation scheme as in 1C. Resected ninth rib similarly seen in superior–posterior aspect of the specimen (yellow arrow).

Immediately after *en bloc* resection and prior to formalin fixation of the cancer specimen, the specimen’s surface topography was virtually captured and reconstructed *ex vivo* using a commercially available structured light handheld 3D scanner (EinScan Pro HD, Shining 3D, Hangzhou, China) and software (EXScan, Shining 3D). The specimen was imaged on each surface until all data were captured yielding one “point cloud” of each specimen surface. Using the EXScan software, these datasets were aligned by selecting three corresponding landmarks on each point cloud to form a cohesive model, which was then meshed into a watertight, high-fidelity virtual 3D model of the resection specimen (Fig. 1A, B). Following completion of the 3D scan, the specimen was processed per standard of care pathologic processing. 3D scanning was performed in the pathology room following specimen accession, required approximately 20 min to complete, and did not delay standard-of-care pathologic processing.

### Virtual 3D specimen mapping

The specimen model was uploaded into a custom computer-aided design (CAD) software as a .ply file, allowing for virtual annotation which took place the day following surgery. Alongside the pathology prosector, a research assistant mirrored the standard-of-care grossing process by virtually annotating the model within the CAD software as the physical specimen underwent standard-of-care inking, sectioning, and processing, thus creating a virtual replica of pathological processing (Fig. 1C, D). Inked surfaces were replicated virtually using a coloring feature while cut sections were demarcated with solid white lines. Perpendicular sections were visually distinguished using color coding, and shave sections were noted using the brush tool. Each section submitted for histologic analysis was labeled with a letter corresponding to the cassette listed within the final pathology report. The pathology team member then verified the accuracy of the 3D specimen map. The final map was exported as a .zip file, which was collated with the original unmapped model and was then converted into a .glb file format. Final pathology demonstrated negative margins; therefore, AR was not used to guide re-excision in this index case. Virtual annotation was performed alongside routine grossing and did not interrupt or delay standard-of-care pathologic processing.

### Augmented reality integration and preoperative use

**FIG. 2.**
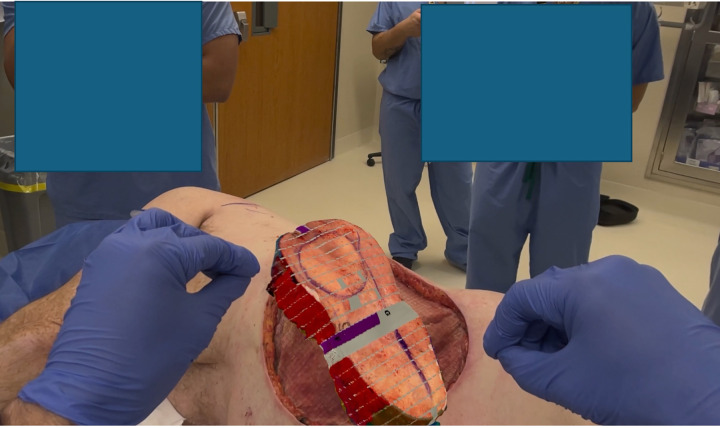
First-person view through the Apple Vision Pro (AVP) headset. Surgeon reimplantation of the mapped holographic left chest wall specimen into the resection bed during staged reconstruction using the AVP augmented reality headset. Inked surfaces and submitted sections on the specimen map were used to accurately orient the specimen.

AR visualization was performed using the Apple Vision Pro (AVP) headset (Apple Inc, Cupertino, CA, 2024). The .glb file of the annotated specimen map was uploaded to the AVP using the Holomedicine Preview 3 D model viewer (apoQlar Medical, Hamburg, Germany). During the preoperative setup, the research team met with the orthopedic oncologic surgeon who performed the initial resection in the operating room. Prior to sterile draping and skin preparation, using the AVP, the surgeon holographically projected the mapped model into space and reoriented it into the resection bed. Given that this surgeon was a first-time AR user, instruction and orientation to the headset were provided by the team in real time. This process was captured via the AVP’s screen record function (Fig. 2).

On the day of reconstruction, 4 days after the oncologic resection, the research team, faculty orthopedic oncologic surgeon, and orthopedic oncology fellow were present in the operating room during preoperative set-up. Both the faculty surgeon and fellow were able to properly orient and resize the model back into the resection bed within the preoperative set-up time. During initial resection, partial rib excision was performed and, using this specimen characteristic, which was also present on the model, holographic alignment into the resection bed was confirmed. Additionally, the inked surfaces of the mapped model were correlated with their known orientations. The entire AR-related workflow, from entering and exiting the operating room, took less than 10 min, required no patient contact, and did not interrupt the standard preoperative setup. Surgeon orientation to the AVP and its features took 5 min, and reorientation of the specimen took an additional 5 min.

## Discussion

In this proof-of-concept single-case study, we demonstrate the feasibility of using AR to reorient a 3D-scanned, virtually mapped tumor model into the resection bed without disrupting routine patient care. The workflow was coordinated with standard specimen handling (prior to formalin fixation) and operative steps; first-time users required a 5-min onboarding to the AVP headset and AR model placement with verification required an additional 5 min, suggesting ready transferability. Despite being a single case, this approach appears feasible and replicable for intraoperative localization tasks.

This study advances prior 3D modeling and margin communication workflows described by our team, focusing on postoperative mapping and reuse of the mapped model during planned reresection.^8^ While our prior work focused on creating and annotating specimen-derived 3D models for postoperative margin communication and later reference, to our knowledge, this is the first report of AR-based reregistration of a specimen-derived model into the patient’s wound bed at a subsequent operation as a framework for potential margin relocalization. The novelty of this case lies in the end-to-end demonstration of a clinically compatible pipeline performed without disrupting routine pathology processing or operative workflow in a radiated, previously resected chest wall sarcoma wound bed undergoing staged reconstruction, where anatomic landmarks are particularly altered. In this context, AR provides a surgeon-facing, spatially intuitive interface to communicate specimen orientation and margin information efficiently. Although this index case did not have a positive margin, we detail a stepwise workflow that could support AR relocalization of a margin within the resection bed, especially relevant when early wound healing prior to reconstruction alters native anatomic landmarks of the resection bed. Relocalizing soft tissue points has been shown to be challenging, with one head and neck study showing greater than 1 cm of mismatch in 32% of attempts to relocate the same soft tissue point within the oropharynx after just 5 min.^7^ Therefore, the use of AR to visualize surgical anatomy may enhance intraoperative orientation and streamline surgical workflow. By enabling *in situ* visualization of the specimen-derived 3D model, we demonstrate a reproducible postoperative mapping and reorientation case using AR to enhance intraoperative orientation and surgical workflow.

Interest in surgical AR has accelerated, with a recent systematic review in orthopedic surgery suggesting utility of AR as a time-saving and accuracy-enhancing device.^10^ Jud et al. demonstrated improved margin accuracy in AR-assisted tumor resections compared with standard-of-care surgery in porcine femoral tumor models, demonstrating a 90.2% probability of achieving a 10 mm margin with AR versus 70.7% via conventional techniques.^10^ While encouraging, this data is derived largely from static anatomy and preclinical settings. To our knowledge, no prior study has examined registration of a specimen-derived holographic model into a human wound bed to support margin relocalization, despite the potential benefit.

This study is limited in that it reports a single case of AR registration of a 3D tumor model without quantitative measurement of accuracy, which limits generalizability. We attempted to mitigate this by standardizing the workflow, using predefined anatomic landmarks for registration, and performing specimen scanning before formalin fixation to reduce specimen distortion. This allowed us to reorient the specimen accurately, to the best of our ability, with relative ease of use. Furthermore, while chest wall respiratory motion and soft tissue deformation upon excision may have driven perceived alignment independent of true orientation accuracy, this study nonetheless demonstrates feasibility in integration of the scanning, mapping, and AR processes into standard pathology and surgical workflows. Repeat studies should assess the replicability of this workflow with different specimen types and across different surgical subspecialties. Additionally, assessing the concordance of specimen-based mapped and resection bed margins in cases with positive margins offers the next step for this technology.

AR-guided surgical decision-making, particularly in the staged reconstructive setting described in this study, may enhance intraoperative spatial anatomy visualization. If validated and adopted on a larger scale, future use of this workflow could improve reresection precision, which could translate into improved oncologic outcomes. Larger-scale, multicenter studies should evaluate this methodology to assess utility, added workload, error rates, and added operative time, with the development of standardized image-to-model pipelines.

## Conclusion

This proof-of-concept case report demonstrates the feasibility of 3D scanning, mapping, and AR-guided projection National Cancer Institute 5K08CA293255-02 of a tumor specimen hologram into the resection bed during staged reconstruction following radical resection of a recurrent undifferentiated pleomorphic sarcoma of the chest wall, providing a workflow framework for margin relocalization if close or positive margins are identified. This methodology was compatible with both surgical and pathology processes, without interrupting or delaying standard-of-care workflows.

## Abbreviations

AR: Augmented Reality
AVP: Apple Vision Pro
MRI: Magnetic Resonance Imaging
CAD: Computer-aided Design
